# 2,9-Di­methyl­phenanthro[2,1-*b*:6,5-*b*′]di­thio­phene

**DOI:** 10.1107/S2414314626007005

**Published:** 2026-07-28

**Authors:** Tetsuji Moriguchi, Misa Sasaki, Noriko Miyoshi

**Affiliations:** a Department of Material Science, Faculty of Engineering, Kyushu Institute of Technology, 1-1 Sensui-cho, Tobata-ku Kitakyushu, Fukuoka, Japan; b Technical Support Department, Management Headquarters, Kyushu Institute of Technology, 1-1 Sensui-cho, Tobata-ku, Kitakyushu 804-8550, Japan; University of Aberdeen, United Kingdom

**Keywords:** crystal structure, heteropolycyclic aromatic compounds, C—H⋯π inter­actions

## Abstract

The title compound, C_20_H_14_S_2_, was synthesized from a dimethyl-substituted thio­phene precursor using an oxidative photocyclization reaction. The mol­ecule has a puckered structure due to steric crowding and the extended structure is consolidated by C—H⋯π inter­actions between columns of stacked mol­ecules.

## Abstract 2

The title mol­ecule is puckered due to steric crowding. The extended structure features C—H⋯π inter­actions.

## Graphical abstract

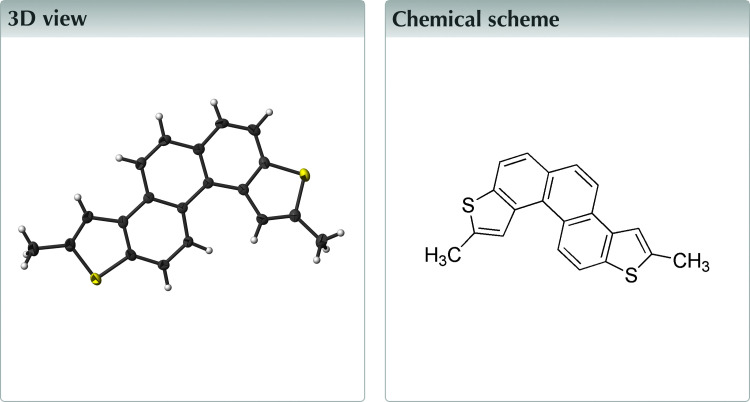

## Structure description

Polycyclic aromatic hydro­carbons, or π-conjugated materials, are an important class of organic compounds that have brought about significant advancements in the field of organic electronics due to their crucial property of conductivity (Wang *et al.*, 2012 ▸). Previously, the authors reported on the synthesis of various types of polycyclic aromatic compounds using extended π-conjugated materials and the evaluation of their organic photoelectronic properties (Moriguchi *et al.*, 2016 ▸). More recently, we also reported on the structural properties and crystal structures of heteropolycyclic aromatic compounds for semiconductor materials using X-ray diffraction analysis (Moriguchi *et al.*, 2017*a* ▸; Moriguchi *et al.*, 2017*b* ▸; Moriguchi *et al.*, 2018 ▸).

**Figure 1:**
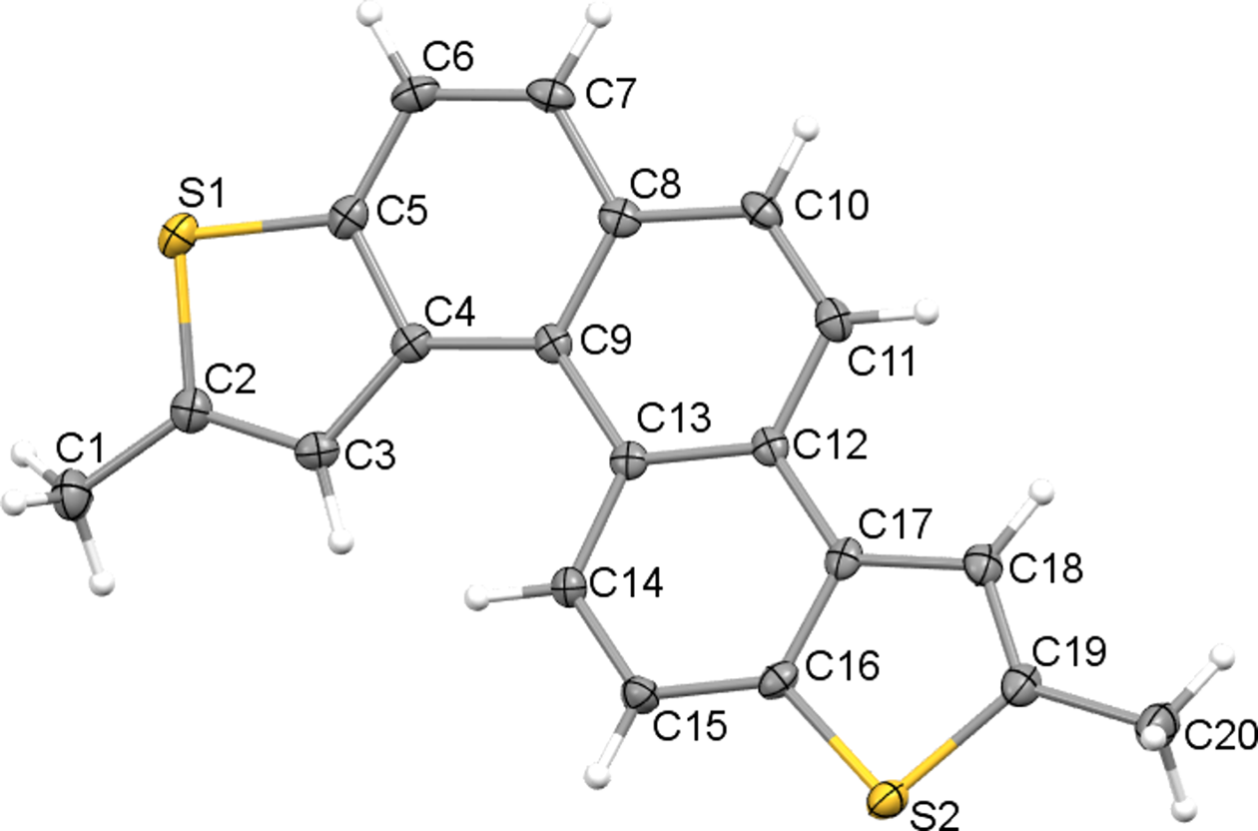
The mol­ecular structure of (**I**), with displacement ellipsoids drawn at the 50% probability level.

**Figure 2:**
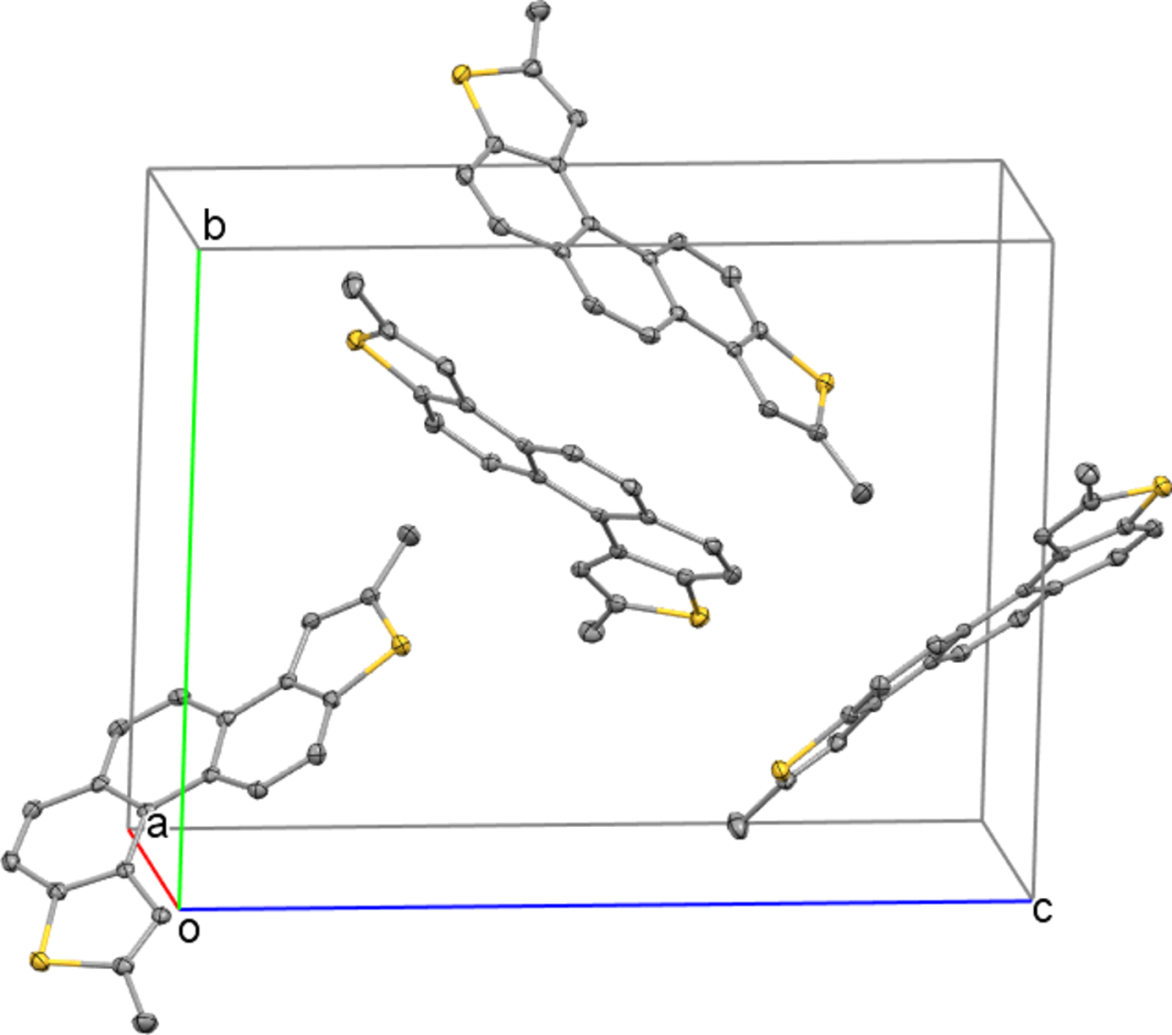
Packing diagram of (**I**), with displacement ellipsoids drawn at the 50% probability level. H atoms have been omitted for clarity.

**Figure 3:**
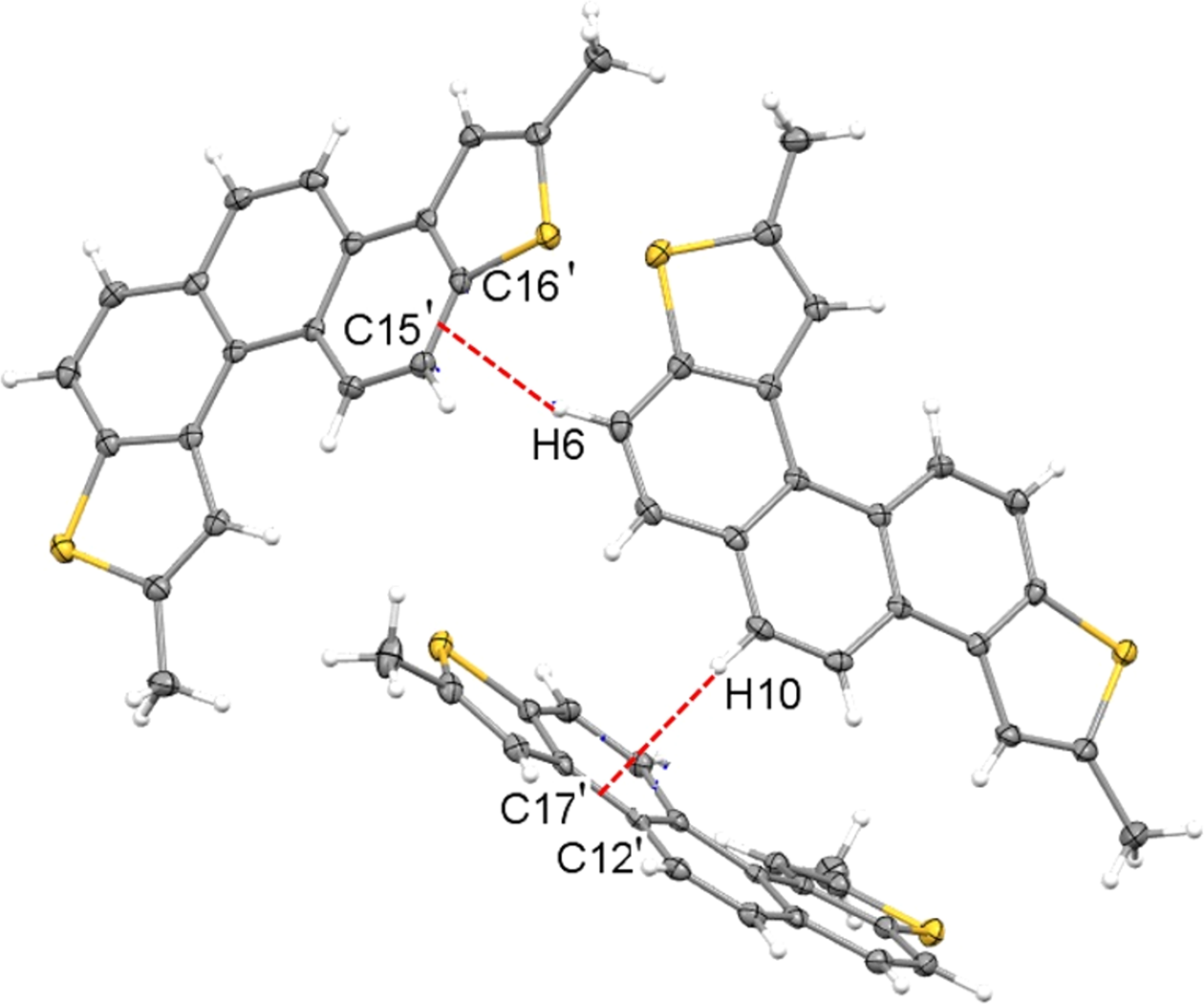
View of the packing of (**I**), showing C—H⋯π inter­actions between adjacent mol­ecules.

As part of our ongoing studies in this area, this article describes the synthesis and structure determination of the title compound, C_20_H_14_S_2_, (**I**). This compound crystallizes in the non-centrosymmetric space group *P*2_1_2_1_2_1_, with one mol­ecule in the asymmetric unit (Fig. 1 ▸). The shape of the mol­ecule is puckered and the dihedral angle between the terminal thio­phene rings (C1–C4/S1 and C16–C19/S2) is 17.26 (9)° (Fig. 3 ▸). The dihedral angle between the C4–C9 and C12–C17 aromatic rings is 11.61 (9)°. This mol­ecular distortion may arise due to intra­molecular congestion: in particular, the short H3⋯H14 contact of 1.98 (3) Å, which is relieved by the mol­ecular twisting. In the extended structure, the mol­ecules are linked by C—H⋯π inter­actions (Table 1 ▸), and are not oriented as a π–π stacked structure (Figs. 2 ▸ and 3 ▸).

## Synthesis and crystallization

All reagents and solvents were purchased from commercial sources and were used without further purification. The electron impact (EI) mass spectrum (MS) of the compound was obtained on a Jeol JMS-SX102A spectrometer using di­chloro­methane (DCM) as the solvent. The instrument was operated in positive ion mode over an *m*/*z* range.

1,3-Bis[2-(2-metyl­thio­phen­yl)ethen­yl]benzene (1.0 mmol) was dissolved in 200 ml of benzene in a round-bottomed flask at room temperature. To this, iodine (10 mmol) was added, the mixture was stirred and irradiated with UV light using a high-pressure Hg lump for 14 h, quenched with 1.0 mol l^−1^ Na_2_S_2_O_3_ solution, allowed to warm to room temperature and extracted twice with AcOEt. The organic layer was washed once with 50 ml water, twice with 50 ml of brine, dried over MgSO_4_ and the solvent was removed under reduced pressure. The title compound was obtained using silica gel (Wako gel C-300) column chromatography (200 mg, 63% yield) with CH_2_Cl_2_ as an eluent. Suitable single crystals of (**I**) in the form of yellow prisms were obtained at room temperature from a solution of di­chloro­methane–*n*-hexane (1/1 *v*/*v*). MS(EI): *M*^+^ 318.

## Refinement

Key crystallographic data are listed in Table 2 ▸. The H atoms were located in difference maps and their positions were freely refined.

## Supplementary Material

Crystal structure: contains datablock(s) I. DOI: 10.1107/S2414314626007005/hb4565sup1.cif

Structure factors: contains datablock(s) I. DOI: 10.1107/S2414314626007005/hb4565Isup2.hkl

Supporting information file. DOI: 10.1107/S2414314626007005/hb4565Isup3.cml

CCDC reference: 2570636

Additional supporting information:  crystallographic information; 3D view; checkCIF report

## Figures and Tables

**Table 1:** Hydrogen-bond geometry (Å, °) Define Cg2/3/5

*D*—H⋯*A*	*D*—H	H⋯*A*	*D*⋯*A*	*D*—H⋯*A*
C1—H1B⋯*Cg*3^i^	1.00 (3)	2.84 (3)	3.714 (3)	147 (2)
C7—H7⋯*Cg*2^ii^	0.91 (2)	2.962 (19)	3.767 (2)	148.3 (15)
C10—H10⋯*Cg*5^ii^	0.99 (2)	2.960 (19)	3.427 (2)	110.1 (13)

Symmetry codes: (i) 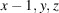; (ii) 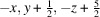.

**Table 2:** Experimental details

Crystal data	
Chemical formula	C_20_H_14_S_2_
*M*_r_	318.47
Crystal system, space group	Orthorhombic, *P*2_1_2_1_2_1_
Temperature (K)	90
*a*, *b*, *c* (Å)	6.5043 (17), 13.456 (3), 17.082 (4)
*V* (Å^3^)	1495.0 (7)
*Z*	4
Radiation type	Mo *K*α
μ (mm^−1^)	0.35
Crystal size (mm)	0.45 × 0.10 × 0.10

Data collection	
Diffractometer	Bruker SMART APEX CCD
Absorption correction	Multi-scan (*SADABS*; Bruker, 2009 ▸)
*T*_min_, *T*_max_	0.611, 0.745
No. of measured, independent and observed [*I* ≥ 2u(*I*)] reflections	14478, 2666, 2543
*R*_int_	0.037
(sin θ/λ)_max_ (Å^−1^)	0.598

Refinement	
*R*[*F*^2^ > 2σ(*F*^2^)], *wR*(*F*^2^), *S*	0.027, 0.065, 1.08
No. of reflections	2666
No. of parameters	255
H-atom treatment	All H-atom parameters refined
Δρ_max_, Δρ_min_ (e Å^−3^)	0.20, −0.17
Absolute structure	Hooft *et al.* (2010 ▸)
Absolute structure parameter	−0.03 (3)

Computer programs: *APEX2* (Bruker, 2009 ▸), *SAINT* (Bruker, 2009 ▸), *olex2.solve* (Bourhis *et al.*, 2015 ▸), *olex2.refine* (Bourhis *et al.*, 2015 ▸) and *OLEX2* (Dolomanov *et al.*, 2009 ▸).

## full crystallographic data

### 2,9-Dimethylphenanthro[2,1-b:6,5-b']dithiophene Crystal data

**Table:**

C_20_H_14_S_2_	*D*_x_ = 1.415 Mg m^−^^3^
*M**_r_* = 318.47	Mo *K*α radiation, λ = 0.71073 Å
Orthorhombic, *P*2_1_2_1_2_1_	Cell parameters from 6305 reflections
*a* = 6.5043 (17) Å	θ = 2.4–25.1°
*b* = 13.456 (3) Å	µ = 0.35 mm^−^^1^
*c* = 17.082 (4) Å	*T* = 90 K
*V* = 1495.0 (7) Å^3^	Prism, clear light yellow
*Z* = 4	0.45 × 0.10 × 0.10 mm
*F*(000) = 665.274	

### 2,9-Dimethylphenanthro[2,1-b:6,5-b']dithiophene Data collection

**Table:**

Bruker SMART APEX CCD diffractometer	2666 independent reflections
Radiation source: sealed tube	2543 reflections with *I*≥ 2u(*I*)
Graphite monochromator	*R*_int_ = 0.037
Detector resolution: 8 pixels mm^-1^	θ_max_ = 25.1°, θ_min_ = 2.4°
ω scans	*h* = −7→7
Absorption correction: multi-scan SADABS (Bruker, 2009)	*k* = −16→16
*T*_min_ = 0.611, *T*_max_ = 0.745	*l* = −20→20
14478 measured reflections	

### 2,9-Dimethylphenanthro[2,1-b:6,5-b']dithiophene Refinement

**Table:**

Refinement on *F*^2^	Primary atom site location: iterative
Least-squares matrix: full	All H-atom parameters refined
*R*[*F*^2^ > 2σ(*F*^2^)] = 0.027	*w* = 1/[σ^2^(*F*_o_^2^) + (0.0346*P*)^2^ + 0.2686*P*] where *P* = (*F*_o_^2^ + 2*F*_c_^2^)/3
*wR*(*F*^2^) = 0.065	(Δ/σ)_max_ = 0.001
*S* = 1.08	Δρ_max_ = 0.20 e Å^−^^3^
2666 reflections	Δρ_min_ = −0.17 e Å^−^^3^
255 parameters	Absolute structure: Hooft *et al.* (2010)
0 restraints	Absolute structure parameter: −0.03 (3)
0 constraints	

### 2,9-Dimethylphenanthro[2,1-b:6,5-b']dithiophene Fractional atomic coordinates and isotropic or equivalent isotropic displacement parameters (Å^2^)

**Table:**

	*x*	*y*	*z*	*U*_iso_*/*U*_eq_	
S1	−0.04847 (9)	0.63225 (4)	1.13345 (3)	0.02673 (13)	
S2	0.15028 (9)	0.18292 (4)	0.70895 (3)	0.02671 (13)	
C1	−0.3530 (4)	0.68925 (17)	1.02692 (15)	0.0332 (5)	
H1a	−0.377 (4)	0.701 (2)	0.9739 (18)	0.062 (9)*	
H1b	−0.484 (5)	0.660 (2)	1.0463 (16)	0.067 (9)*	
H1c	−0.336 (5)	0.751 (2)	1.0483 (16)	0.068 (9)*	
C20	0.4911 (4)	0.05841 (19)	0.68237 (14)	0.0383 (6)	
H20a	0.628 (5)	0.047 (2)	0.6963 (16)	0.055 (8)*	
H20b	0.424 (5)	−0.007 (2)	0.6861 (16)	0.066 (9)*	
H20c	0.484 (4)	0.0714 (19)	0.6294 (16)	0.055 (8)*	
C2	−0.1704 (3)	0.62557 (14)	1.04301 (11)	0.0245 (4)	
C3	−0.0746 (3)	0.56109 (13)	0.99428 (11)	0.0214 (4)	
H3	−0.121 (3)	0.5547 (14)	0.9429 (11)	0.018 (5)*	
C4	0.1031 (3)	0.51287 (13)	1.02696 (10)	0.0193 (4)	
C5	0.1397 (3)	0.54895 (13)	1.10359 (11)	0.0224 (4)	
C19	0.3958 (3)	0.13846 (14)	0.73123 (11)	0.0264 (5)	
C18	0.4745 (3)	0.18496 (14)	0.79448 (11)	0.0240 (4)	
H18	0.598 (3)	0.1690 (15)	0.8135 (11)	0.020 (5)*	
C17	0.3420 (3)	0.25900 (13)	0.82838 (10)	0.0201 (4)	
C16	0.1569 (3)	0.26534 (13)	0.78785 (10)	0.0215 (4)	
C6	0.3123 (3)	0.52437 (14)	1.14820 (11)	0.0252 (4)	
H6	0.332 (3)	0.5511 (15)	1.2015 (13)	0.030 (6)*	
C7	0.4554 (4)	0.46387 (14)	1.11539 (11)	0.0237 (4)	
H7	0.572 (3)	0.4467 (14)	1.1416 (11)	0.015 (5)*	
C15	−0.0010 (3)	0.32809 (14)	0.81307 (11)	0.0225 (4)	
H15	−0.128 (4)	0.3298 (15)	0.7842 (12)	0.031 (6)*	
C14	0.0304 (3)	0.38536 (13)	0.87894 (11)	0.0208 (4)	
H14	−0.086 (3)	0.4230 (13)	0.8932 (10)	0.013 (5)*	
C10	0.5811 (3)	0.35708 (14)	1.00967 (11)	0.0227 (4)	
H10	0.710 (3)	0.3506 (14)	1.0398 (11)	0.025 (6)*	
C11	0.5574 (3)	0.30801 (14)	0.94137 (11)	0.0222 (4)	
H11	0.655 (3)	0.2674 (15)	0.9225 (11)	0.023 (5)*	
C9	0.2457 (3)	0.44063 (12)	0.99489 (11)	0.0176 (4)	
C13	0.2158 (3)	0.38424 (13)	0.92283 (10)	0.0176 (4)	
C8	0.4253 (3)	0.42170 (13)	1.03969 (10)	0.0205 (4)	
C12	0.3746 (3)	0.31869 (13)	0.89681 (10)	0.0184 (4)	

### 2,9-Dimethylphenanthro[2,1-b:6,5-b']dithiophene Atomic displacement parameters (Å^2^)

**Table:**

	*U*^11^	*U*^22^	*U*^33^	*U*^12^	*U*^13^	*U*^23^
S1	0.0315 (3)	0.0248 (2)	0.0239 (2)	0.0001 (2)	0.0038 (2)	−0.0052 (2)
S2	0.0352 (3)	0.0251 (2)	0.0199 (2)	0.0027 (2)	−0.0002 (2)	−0.0034 (2)
C1	0.0330 (13)	0.0281 (11)	0.0384 (13)	0.0082 (11)	0.0022 (11)	−0.0012 (10)
C20	0.0516 (18)	0.0365 (13)	0.0269 (12)	0.0172 (12)	−0.0013 (11)	−0.0066 (10)
C2	0.0261 (10)	0.0200 (9)	0.0275 (10)	−0.0017 (9)	0.0033 (9)	0.0010 (8)
C3	0.0258 (11)	0.0176 (9)	0.0207 (10)	−0.0041 (8)	−0.0023 (9)	0.0013 (8)
C4	0.0228 (11)	0.0155 (9)	0.0197 (9)	−0.0051 (7)	0.0022 (7)	0.0025 (7)
C5	0.0262 (11)	0.0187 (9)	0.0223 (9)	−0.0043 (8)	0.0045 (9)	−0.0002 (7)
C19	0.0369 (13)	0.0197 (9)	0.0226 (9)	0.0063 (9)	0.0025 (9)	0.0033 (8)
C18	0.0275 (11)	0.0198 (9)	0.0248 (9)	0.0054 (9)	0.0011 (9)	0.0054 (9)
C17	0.0263 (10)	0.0153 (8)	0.0187 (8)	−0.0001 (8)	0.0063 (9)	0.0047 (7)
C16	0.0290 (10)	0.0186 (9)	0.0169 (8)	−0.0026 (8)	0.0018 (9)	0.0003 (7)
C6	0.0322 (12)	0.0233 (9)	0.0202 (10)	−0.0066 (9)	−0.0022 (9)	−0.0002 (8)
C7	0.0239 (10)	0.0220 (10)	0.0251 (10)	−0.0033 (9)	−0.0076 (9)	0.0060 (8)
C15	0.0191 (11)	0.0265 (10)	0.0218 (9)	−0.0018 (8)	−0.0012 (8)	−0.0004 (8)
C14	0.0195 (9)	0.0200 (9)	0.0229 (9)	0.0014 (8)	0.0013 (8)	−0.0007 (8)
C10	0.0210 (11)	0.0200 (9)	0.0271 (10)	0.0006 (8)	−0.0028 (9)	0.0068 (8)
C11	0.0219 (10)	0.0161 (9)	0.0286 (10)	0.0012 (9)	0.0026 (9)	0.0038 (8)
C9	0.0183 (9)	0.0133 (8)	0.0212 (9)	−0.0028 (7)	0.0020 (8)	0.0049 (7)
C13	0.0198 (10)	0.0147 (9)	0.0184 (9)	−0.0024 (7)	0.0027 (7)	0.0049 (7)
C8	0.0241 (11)	0.0146 (9)	0.0227 (9)	−0.0025 (8)	−0.0001 (8)	0.0060 (7)
C12	0.0192 (9)	0.0156 (8)	0.0205 (8)	−0.0015 (8)	0.0030 (8)	0.0061 (8)

### 2,9-Dimethylphenanthro[2,1-b:6,5-b']dithiophene Geometric parameters (Å, º)

**Table:**

S1—C2	1.739 (2)	C18—C17	1.439 (3)
S1—C5	1.736 (2)	C17—C16	1.392 (3)
S2—C19	1.747 (2)	C17—C12	1.434 (2)
S2—C16	1.7459 (18)	C16—C15	1.397 (3)
C1—H1a	0.93 (3)	C6—H6	0.99 (2)
C1—H1b	1.00 (3)	C6—C7	1.358 (3)
C1—H1c	0.92 (3)	C7—H7	0.91 (2)
C1—C2	1.490 (3)	C7—C8	1.426 (3)
C20—H20a	0.94 (3)	C15—H15	0.96 (2)
C20—H20b	0.99 (3)	C15—C14	1.379 (3)
C20—H20c	0.92 (3)	C14—H14	0.941 (19)
C20—C19	1.497 (3)	C14—C13	1.420 (3)
C2—C3	1.354 (3)	C10—H10	0.99 (2)
C3—H3	0.931 (19)	C10—C11	1.349 (3)
C3—C4	1.438 (3)	C10—C8	1.431 (3)
C4—C5	1.416 (3)	C11—H11	0.90 (2)
C4—C9	1.451 (3)	C11—C12	1.419 (3)
C5—C6	1.396 (3)	C9—C13	1.459 (3)
C19—C18	1.349 (3)	C9—C8	1.419 (3)
C18—H18	0.89 (2)	C13—C12	1.429 (2)
			
C5—S1—C2	91.55 (10)	C12—C17—C16	119.94 (17)
C16—S2—C19	91.54 (9)	C17—C16—S2	111.49 (14)
H1b—C1—H1a	105 (2)	C15—C16—S2	127.14 (16)
H1c—C1—H1a	104 (2)	C15—C16—C17	121.29 (17)
H1c—C1—H1b	109 (2)	H6—C6—C5	121.4 (13)
C2—C1—H1a	114.3 (17)	C7—C6—C5	117.89 (18)
C2—C1—H1b	112.8 (16)	C7—C6—H6	120.7 (13)
C2—C1—H1c	111 (2)	H7—C7—C6	121.3 (12)
H20b—C20—H20a	105 (2)	C8—C7—C6	121.2 (2)
H20c—C20—H20a	109 (2)	C8—C7—H7	117.4 (12)
H20c—C20—H20b	102 (2)	H15—C15—C16	119.1 (12)
C19—C20—H20a	111.5 (17)	C14—C15—C16	118.73 (18)
C19—C20—H20b	115.3 (17)	C14—C15—H15	122.1 (12)
C19—C20—H20c	113.0 (16)	H14—C14—C15	113.2 (11)
C1—C2—S1	119.84 (15)	C13—C14—C15	123.40 (18)
C3—C2—S1	111.69 (15)	C13—C14—H14	123.3 (11)
C3—C2—C1	128.44 (19)	C11—C10—H10	120.3 (11)
H3—C3—C2	119.4 (12)	C8—C10—H10	117.9 (11)
C4—C3—C2	114.84 (17)	C8—C10—C11	121.75 (19)
C4—C3—H3	125.6 (12)	H11—C11—C10	121.9 (13)
C5—C4—C3	109.83 (17)	C12—C11—C10	120.67 (19)
C9—C4—C3	132.02 (16)	C12—C11—H11	117.5 (13)
C9—C4—C5	118.10 (17)	C13—C9—C4	125.57 (17)
C4—C5—S1	111.97 (15)	C8—C9—C4	116.27 (16)
C6—C5—S1	124.00 (14)	C8—C9—C13	118.12 (16)
C6—C5—C4	123.95 (18)	C9—C13—C14	123.58 (17)
C20—C19—S2	120.21 (17)	C12—C13—C14	117.14 (16)
C18—C19—S2	111.24 (15)	C12—C13—C9	119.14 (16)
C18—C19—C20	128.6 (2)	C10—C8—C7	118.00 (18)
H18—C18—C19	121.4 (13)	C9—C8—C7	122.06 (18)
C17—C18—C19	114.60 (19)	C9—C8—C10	119.93 (17)
C17—C18—H18	124.0 (13)	C11—C12—C17	120.30 (17)
C16—C17—C18	111.12 (17)	C13—C12—C17	119.49 (17)
C12—C17—C18	128.86 (19)	C13—C12—C11	120.08 (16)
			
S1—C2—C3—C4	0.09 (16)	C19—C18—C17—C16	0.72 (19)
S1—C5—C4—C3	3.52 (15)	C19—C18—C17—C12	177.42 (15)
S1—C5—C4—C9	−178.57 (12)	C18—C17—C16—C15	176.11 (15)
S1—C5—C6—C7	−175.12 (16)	C18—C17—C12—C11	−0.5 (2)
S2—C19—C18—C17	−0.14 (16)	C18—C17—C12—C13	−176.35 (18)
S2—C16—C17—C18	−0.96 (15)	C17—C16—C15—C14	0.8 (2)
S2—C16—C17—C12	−177.99 (11)	C17—C12—C11—C10	−173.85 (17)
S2—C16—C15—C14	177.40 (16)	C17—C12—C13—C14	0.78 (18)
C1—C2—C3—C4	178.1 (2)	C17—C12—C13—C9	176.65 (15)
C20—C19—C18—C17	−179.9 (2)	C16—C15—C14—C13	0.1 (2)
C2—C3—C4—C5	−2.33 (19)	C6—C7—C8—C10	−178.76 (18)
C2—C3—C4—C9	−179.85 (15)	C6—C7—C8—C9	0.1 (2)
C3—C4—C5—C6	−173.39 (14)	C7—C8—C10—C11	174.71 (17)
C3—C4—C9—C13	−12.7 (2)	C7—C8—C9—C13	−172.10 (16)
C3—C4—C9—C8	169.6 (2)	C15—C14—C13—C9	−176.58 (17)
C4—C5—C6—C7	1.4 (2)	C15—C14—C13—C12	−0.9 (2)
C4—C9—C13—C14	−7.2 (2)	C14—C13—C9—C8	170.49 (17)
C4—C9—C13—C12	177.26 (17)	C14—C13—C12—C11	−175.03 (16)
C4—C9—C8—C7	5.76 (19)	C10—C11—C12—C13	1.9 (2)
C4—C9—C8—C10	−175.41 (15)	C10—C8—C9—C13	6.72 (19)
C5—C6—C7—C8	−3.8 (2)	C11—C12—C13—C9	0.84 (19)

### 2,9-Dimethylphenanthro[2,1-b:6,5-b']dithiophene Hydrogen-bond geometry (Å, º)

**Table:**

*D*—H···*A*	*D*—H	H···*A*	*D*···*A*	*D*—H···*A*
C1—H1B···*Cg*3^i^	1.00 (3)	2.84 (3)	3.714 (3)	147 (2)
C7—H7···*Cg*2^ii^	0.91 (2)	2.962 (19)	3.767 (2)	148.3 (15)
C10—H10···*Cg*5^ii^	0.99 (2)	2.960 (19)	3.427 (2)	110.1 (13)

Symmetry codes: (i) *x*−1, *y*, *z*; (ii) −*x*, *y*+1/2, −*z*+5/2.
